# Potential Synergistic Supplementation of NAD+ Promoting Compounds as a Strategy for Increasing Healthspan

**DOI:** 10.3390/nu15020445

**Published:** 2023-01-14

**Authors:** Arastu Sharma, Sophie Chabloz, Rebecca A. Lapides, Elisabeth Roider, Collin Y. Ewald

**Affiliations:** 1 Laboratory of Extracellular Matrix Regeneration, Department of Health Sciences and Technology, Institute of Translational Medicine, ETH Zürich, 8603 Schwerzenbach, Switzerland; 2 AVEA Life AG, Bahnhofplatz, 6300 Zug, Switzerland; 3 Department of Dermatology, University Hospital of Basel, 4031 Basel, Switzerland; 4 Robert Larner, MD College of Medicine at the University of Vermont, Burlington, VT 05405, USA; 5 Cutaneous Biology Research Center, Massachusetts General Hospital, Harvard Medical School, Charlestown, MA 02129, USA; 6 Maximon AG, Bahnhofplatz, 6300 Zug, Switzerland

**Keywords:** aging, longevity, supplements, geroprotectors, SIRT1, NAD+, NMN, resveratrol, nutraceuticals, age-related diseases, flavonoids, senolytics, healthspan

## Abstract

Disrupted biological function, manifesting through the hallmarks of aging, poses one of the largest threats to healthspan and risk of disease development, such as metabolic disorders, cardiovascular ailments, and neurodegeneration. In recent years, numerous geroprotectors, senolytics, and other nutraceuticals have emerged as potential disruptors of aging and may be viable interventions in the immediate state of human longevity science. In this review, we focus on the decrease in nicotinamide adenine dinucleotide (NAD+) with age and the supplementation of NAD+ precursors, such as nicotinamide mononucleotide (NMN) or nicotinamide riboside (NR), in combination with other geroprotective compounds, to restore NAD+ levels present in youth. Furthermore, these geroprotectors may enhance the efficacy of NMN supplementation while concurrently providing their own numerous health benefits. By analyzing the prevention of NAD+ degradation through the inhibition of CD38 or supporting protective downstream agents of SIRT1, we provide a potential framework of the CD38/NAD+/SIRT1 axis through which geroprotectors may enhance the efficacy of NAD+ precursor supplementation and reduce the risk of age-related diseases, thereby potentiating healthspan in humans.

## 1. Introduction

The doubling of life expectancy in the last century can be attributed to progressive leaps in health and clinical care in the human population. However, at the expense of longevity comes the increased risk of disease and other physical ailments. Being the main risk factor for the ever-increasing incidence of neurodegeneration, cardiovascular disease, cancer, and other medical ailments, aging has come to the forefront as a new clinical target within itself, aiming to serve as a preventative measure for developing diseases [1]. Aging can be characterized as universal, intrinsic, progressive, and deleterious to the health of an individual. A multimodal onslaught of the body’s physiological processes may be the culprit, whether it be through environmental damage, a decline in endogenous protective mechanisms, or a pure breakdown of the physiological process due to wear and tear [2]. Numerous pathways are also implicated and may break down or become dysfunctional due to aging, resulting in neurodegenerative disorders, as an example [3]. The societal burden of these diseases and age-related pathologies is ever-compounding and must be addressed with novel interventions to improve quality of life for the aging population. The conundrum of aging and the associated risk of developing age-related diseases has the propensity to affect every individual and is further affected and potentially accelerated by other biological and environmental risk factors.

Targeting the hallmarks of aging exists as a model which would allow for interventions ameliorating these processes: genomic instability, telomere attrition, epigenetic alterations, loss of proteostasis, deregulated nutrient sensing, mitochondrial dysfunction, cellular senescence, stem cell exhaustion, and altered intercellular communication. Each intervention is thought to focus on these hallmarks or even have a multimodal crossover effect ameliorating the biological processes of aging [4]. Appropriate, widespread, and easily executable interventions are key factors in combating the newly defined epidemic of aging and age-related disorders. In the field of orthomolecular medicine, the use of supplementation as a therapeutic approach to slow the process of aging is highly applicable when selecting and formulating geroprotective treatments. Longevity supplements have shown exciting potential for improving healthspan, and research into longevity supplements is one of the most rapidly advancing fields in aging science today. However, further human studies are still warranted [5]. Human trials utilizing regenerative or senolytic compounds to target skin aging, in particular, have shown improvement in the biological response to environmental stressors, such as UV and oxidative damage, which often accelerate the process of aging [6,7,8]. Age-related deficits in cognition also arise as viable targets of longevity boosting and geroprotective supplementation. These deficits can be attributed to excessive neuroinflammation, oxidative damage, and metabolic disruption. Nutraceutical interventions, particularly those targeting these numerous disruptive pathways, have shown promise in ameliorating age-related cognitive decline [9]. Administration of combinations of micronutrients and other antioxidant compounds has also shown promising results in promoting longevity and slowing accelerated aging in mice [10]. One compound in particular, nicotinamide adenine dinucleotide (NAD+), is relevant in many biological processes as a cofactor for crucial metabolic functions. These sensitive pathways are interrelated with sirtuins, a family of signaling proteins involved in metabolic regulation, and PARP1, an enzyme that detects DNA damage and facilitates repair pathways. These may be disrupted by external factors and/or intrinsic breakdown due to aging, leading to an increased risk for many disorders, cognitive decline associated with aging, and neurodegenerative disorders such as Alzheimer’s and Parkinson’s disease [11]. Multiple cohort studies spanning various age groups have shown significant decreases in NAD+ levels with age. This dysregulation identifies NAD+ as a potential target for age-related pathologies [12]. In model organisms, NAD+ precursor administration has resulted in delayed muscle atrophy, improved neurodegenerative pathologies [13], and restored metabolic function [14]. The clinically relevant evidence regarding the targeting of NAD+ to replenish endogenous stores and invigorate the NAD+ salvage pathway is compounding, raising the question of how NAD+ administration may be able to affect aging or age-related deficits in humans. Given the importance of maintaining NAD+ levels for optimal health and longevity, restoring NAD+ levels is a promising and feasible approach in promoting health during aging.

Here, we review NAD+ metabolism and its molecular interactions with other longevity-promoting pathways. Numerous studies have analyzed the effects of oral NAD+ precursor administration on specific disease states, muscle performance, metabolic processes, and other metrics. However, the longevity-promoting effect of NAD+ precursor administration can be further optimized and enhanced by the supplementation of NAD+ enhancing and supporting compounds [15]. The longevity-boosting potential of repurposed drugs, such as rapamycin and metformin, has been discussed. However, as data from clinical trials are either lacking or only very sparsely available, they cannot be recommended for clinical routine. Ingredients show promise in the context of longevity, but require further research on their synergistic effects on the NAD+/SIRT1/CD38 axis [16]. With no proven pharmaceutic options available, we will discuss other potent ingredients and nutraceuticals that may influence and modulate the NAD+ axis involving SIRT1 and CD38 in biological mechanisms, to offer further geroprotection by combating the aging process and, additionally, providing each of the nutraceutical’s own health benefits.

## 2. NAD+ Homeostasis

Nicotinamide adenine dinucleotide (NAD+) is one of the primary coenzymes in metabolic processes and is involved with numerous other pathways, such as energy expenditure, metabolic and stress adaptations, and circadian rhythm maintenance. NAD+ levels sharply decline with age, and this decline can be attributed to the activity of CD38, an enzyme responsible for the degradation of NAD+, which disrupts the NAD+ synthesis pathways during the course of aging [17]. NAD+ homeostasis in the body is critical for optimal biological function. NAD+ consuming enzymes function to convert NAD+ to nicotinamide (NAM) and occupy a specific role in biological aging pathology. They are implicated as interventional targets for geroprotection, such as the enzyme CD38, the sirtuins (SIRT) deacetylases, poly [ADP-ribose] polymerase 1 (PARP1: involved in the DNA damage response (DDR)), and the neuronal degenerating and NAD+ draining factor SARM1 [18,19]. NAD+ can be synthesized de novo from nicotinic acid (NA), nicotinamide riboside (NR), and nicotinamide mononucleotide (NMN) or can be salvaged through the NAD+ salvage pathway, which is crucial for recycling the metabolites of biochemical reactions to replete NAD+ stores in the body [20]. An extracellular conversion of NMN to NR by CD73, a cell surface enzyme, represents another mechanism by which the intracellular NAD+ content is maintained [21].

Cellular levels of NAD+ are also regulated by the enzyme nicotinamide N-methyltransferase (NNMT). When NNMT methylates nicotinamide, creating methylnicotinamide (MNT), the amount of free nicotinamide is reduced and, therefore, not available for conversion into NAD+ through the NAD+ salvage pathway. NNMT and MNT have been associated with obesity and diabetes mellitus type two [22]. However, NNMT also has been shown to stabilize SIRT1, thereby exerting beneficial metabolic effects and protecting against oxidative stress-induced cellular injury [23,24]. Methylnicotinamide has also been shown to increase lifespan [25]. However, many NNMT inhibitors have been developed, and promising data establish a potential use for these in the treatment of pathologic states including, but not limited to, cancer, obesity, metabolic disorders, and alcohol-related fatty liver disease [22,26,27,28,29]. Taken together, the interaction between NNMT, MNT, and the pathways they help regulate plays a significant role in NAD+ homeostasis and, thus, the complex disease states that undoubtedly influence the aging process.

### 2.1. NAD+, Sirtuins and Longevity-Promoting Pathway

Disruption of proper NAD+ and loss of protective sirtuin activity have emerged as the prime targets for NAD+-based interventions [30]. Administration of NAD+ precursors, namely NR and NMN, has been shown to alleviate age-related NAD+ pathology, particularly in the context of age-related diseases [13,31,32]. Aging has been associated with a decreased NAD+/NADH ratio in human plasma through the deterioration of NAD+ stores, rather than an increase in NADH [12]. Replenishment of NAD+ rescues mitochondrial regulatory function from NAD+ induced pseudohypoxic mitochondrial stress during aging [14].

**Figure 1:**
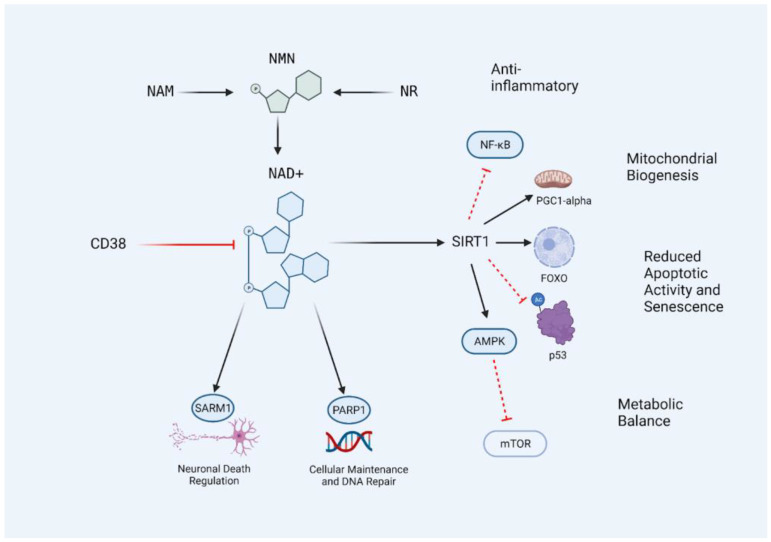
The CD38/NAD+/SIRT1 Axis. NAD+ levels in the body can be influenced by the supplementation of precursors nicotinamide (NAM), nicotinamide riboside (NR), and nicotinamide mononucleotide (NMN). NAD+ levels decrease with age and are further metabolized by the activation of SIRT1, PARP1, SARM1, and CD38. Restoring NAD+ levels allows for an increase in SIRT1 activity due to increased substrate availability, resulting in the inhibition of age-promoting pathways and activation of adaptive and protective transcription factors and processes. The central lineage may be described as the CD38/NAD+/SIRT1 axis, and targeting this access with nutraceutical interventions may prevent the age-related decline of NAD+ levels in the body. Black lines indicate conversion or activation. Red lines indicate inhibitors or destroyers of the indicated target.

SIRT1, a member of a protein family that is involved in the cellular response to various stressors, has been shown to be implicated in longevity, but experiments and analysis have yielded mixed and context-dependent results. Nonetheless, high-level athletes exhibit higher telomere length and lowered insulin resistance, correlating with higher levels of SIRT1 expression [33]. The beneficial activity of SIRT1 may rely on the deacetylation and subsequent activation of the Forkhead transcription factors FoxO and PGC1α [34,35]. FoxOs is a transcription factor involved in stress resistance, cell cycle arrest, apoptosis, and tumor suppression. Activation of FoxOs has been linked with longevity in worms and flies [36,37]. Important processes, including growth, development, metabolism, reproduction, and longevity, are regulated through insulin/insulin-like growth factor signaling (IIS) working through the activity of FoxO. This pathway has been shown to extend neuronal activity and longevity under low IIS conditions [38,39]. PGC1α exerts its influence on mitochondrial biogenesis, where deficits are apparent in metabolic disease states. Overexpression of PGC1α has shown improved insulin sensitivity in muscle [40,41,42]. AMPK, involved in energy expenditure, also exhibits similarity to and bi-directional innervation from SIRT1 and further inhibits mTOR, an inhibitory process that has also been linked to longevity. It further activates SIRT1 by increasing available NAD+ stores [43]. Furthermore, nuclear factor κB (NF-κB) signaling, a process involved in innate immunity, can be inhibited by SIRT1 activity to reduce prolonged inflammatory signaling [44]. Depending on NAD+ stores in the body, SIRT1 activity arises as an interesting target in age-related pathway manipulation to promote longevity (Figure 1) [31,45,46,47]. The importance of maintaining an adequate NAD+ level for optimal SIRT1 activity during aging may be a key factor in regulating longevity.

### 2.2. NAD+ and Circadian Rhythm

NAD+ stores in the body are extremely crucial for the programming of the circadian metabolic clock. Older mice with less abundant NAD+ experienced prolonged repression of CLOCK/BMAL1 transcription compared to younger mice with more abundant NAD+, resulting in disrupted and dampened mitochondrial and transcriptional oscillation [48]. NAD+ supplementation and restoration in circadian mutant mice have also been shown to re-establish proper respiratory oscillations and circadian metabolic regulation, particularly through SIRT3 regulatory activity [49]. An abundant reserve of NAD+ and proper sirtuin activation are crucial for maintaining the integrity of the various endogenous clocks, and supplementation with NAD+ precursors may potentially alleviate any age-related perturbations in these circadian processes [50]. NAD+ deficiencies are observed in these numerous age-related diseases, and NAD+-based interventions are currently underway in an attempt to ameliorate this common conundrum these diseases share [51,52,53,54].

### 2.3. CD38 and the Decline of NAD+

Adequate intracellular levels of NAD+ are crucial for the proper function of several biological processes, including mitochondria metabolism [55]. In mitochondrial disease models, PARP inhibition or NR supplementation ameliorated deficits in metabolism and exercise capacity [56]. Strikingly, a cell surface glycoprotein, cluster of differentiation 38 (CD38), also acts as one of the main consumers of NAD+ stores in the body and is involved in immune activation and inflammatory signaling [57]. The NAD+ degrading enzyme CD38 has also been shown to regulate SIRT1 activity along with NAD+ availability [58]. CD38 expression increases during aging and CD38 knockout models exhibit heightened NAD+ levels and protection against obesity, metabolic disorders, and cancer progression [59], illuminating CD38′s role in increasing the risk of developing age-related metabolic diseases [60]. It has been suggested that CD38 deficiency may result in susceptibility to autoimmunity and decreased immune integrity, but further studies in humans are required to analyze the effects of insufficient CD38 [61]. Nevertheless, reducing chronic inflammation is an aim of healthy aging; inflammatory profiles of senescent cells are associated with heightened CD38 activation and resulting NAD+ degradation through compounding aging and inflammation processes [62]. Interestingly, this decline in NAD+ and increase in CD38 with age can potentially be attributed to another age-related phenomenon: the accumulation of senescent cells, cells that exhibit permanent growth arrest. Inflammation and aging, or “inflammaging,” describes the build-up of inflammatory signaling compounds due to cellular senescence and the expression of CD38 as a senescent cellular inflammatory profile. Senescent cells, through their senescence-associated secretory profiles (SASP) of a milieu of IL-6, TNF-α, and CXCL 1 and 2, induce CD38 expression in M1 macrophages, causing age-related NAD+ decline in non-senescent phenotypic populations. These age-related senescent cells have also been shown to accumulate in hepatic and visceral white adipose tissue, and the resulting SASP induces pro-inflammatory M1 macrophage proliferation and enhanced CD38 expression [63,64,65]. The significant interplay between SASP, CD38, and NAD+ levels reveals an interventional target to further enhance available NAD+ in the body, particularly through strong inhibition of CD38 activity or expression well into the aging process to prevent any age-related biological deficits.

### 2.4. NMN as an NAD+ Boosting Therapeutic

When using an interventional method to target declining NAD+ levels, the bioavailability and proper uptake of the proposed supplement must be analyzed. NAD+ precursors, such as NR, NMN, and NAM, have been implicated in the adequate uptake and subsequent biosynthesis of NAD+ [66,67]. For instance, NAD3^®^, an NR-based dietary supplement, was shown in a clinical trial in older adults to significantly improve NAD+ levels as well as improve blood lipid profiles by decreasing LDL:HDL cholesterol ratios [68]. Another meta-analysis revealed that NAD+ boosting mechanisms are successful in decreasing total cholesterol, triglyceride, and the LDL:HDL ratio [69]. Moreover, NMN in particular serves as a molecule of interest in the context of NAD+ support, mainly due to the fact that only one conversion step is required to reach NAD+, compared to more required steps for other precursors. NMN supplementation has had numerous benefits, ranging from cardiovascular to neurodegenerative disease contexts, and proper uptake and resulting biosynthesis of NAD+ is observed in varying tissue types [70,71,72]. A successful increase in intracellular NAD+/NADH ratio was evident in a clinical trial in adults aged 40 to 65 years over 60 days at a dosage of 300 mg daily, providing key safety and efficacy data to support the use of NMN Uthever^TM^ as a valid interventional approach to increase NAD+ in a safe manner [73]. NMN supplementation has also been shown to target one of the hallmarks of aging, telomere attrition. Short term supplementation of NMN in pre-aging mice and humans, ages 45 to 60 years, showed a significant increase in telomere length in peripheral blood mononuclear cells, as well as altered fecal microbiota benefiting immune and metabolic pathways [74], where specific administration in mice has been shown to increase the ratio of beneficial to harmful gut bacteria strains [75]. NMN supplementation has also been shown to enhance the miRNA vascular expression profile in aged mice [76]. Respiratory parameters and aerobic capacity can also be enhanced through six weeks of NMN supplementation in adult runners, likely attributed to the enhanced oxygen utilization occurring in skeletal muscle as a result of increased NAD+ availability [77]. Furthermore, daily NMN supplementation at 250 mg was shown to be well tolerated, efficacious when increasing the NAD+/NADH ratio, and improved muscle function in aged but otherwise healthy men [78]. NMN supplementation has also been implicated in improving circadian rhythm patterns in older adults, where 250 mg daily for 12 weeks led to improvements in sleep quality, as measured through PSQI, fatigue, and physical performance [79].

Nevertheless, caution is warranted, as more recent data showed that in response to NR administration, increased rates of brain metastasis were observed after intracardial injection of triple-negative breast cancer (TNBC) cells in 9 out of 11 NR-treated mice compared to 3 out of 12 mice treated controls, proposing the increased risk of cancer metastasis in response to NR administration [80]. A contrasting study, however, showed no adverse effects on rats after administration of NMN at doses of 375, 750, and 150 mg/kg/d, with a maximum acute dose identified at 2666 mg/kg/d, enhancing the safety profile of NMN supplementation [81] To understand if there are differences in the safety of NAD+ precursor use, additional research is needed. To help to understand the translatability and mechanistic actions described in animal models, several clinical trials are ongoing (Table 1).

### 2.5. A Combination Approach for Restoring Youthful NAD+ Levels during Aging

As the interest in geroprotective trends grows, the momentum of clinical trials utilizing NAD+ boosting molecules is increasing. However, proper metrics evaluating the benefits and efficacy of intervention of NAD+ boosting supplements must be further standardized to offer significant and informative comparative effects of geroprotective interventions. NAD+ precursors have shown promising effects in human trials (Table 1), but with the involvement of CD38 and SIRT1 pathways, many potential interactions exist to further amplify the NAD+ boosting effects of orthomolecular interventions. SIRT1 activation, NAD+ synergy, CD38 inhibition, and methylation support are all important factors when considering maximizing the potential of NAD+ precursor administration. The synergistic effects and health benefits of the compounds involved in these three processes will be further discussed to create a foundation to pave the way for potentiating supplement formulations for maximal NAD+ boosting effects. One approach to maximize NAD+ boosting capacity would be to supplement safe levels of NMN with other geroprotectors and nutraceuticals that will further enhance endogenous NAD+ levels, ensuring the restoration of physiological levels of NAD+. Therefore, we summarized below different geroprotectors that enhance endogenous NAD+ levels but also have their own longevity benefits. For a selection of the following compounds, synergistic health effects of NMN and a geroprotector have been reported, suggesting the benefit of this combinatorial approach.

## 3. Potential Synergistic Nutraceutical Interventions

### 3.1. Stilbenes: Resveratrol and Pterostilbene

**Figure 2:**
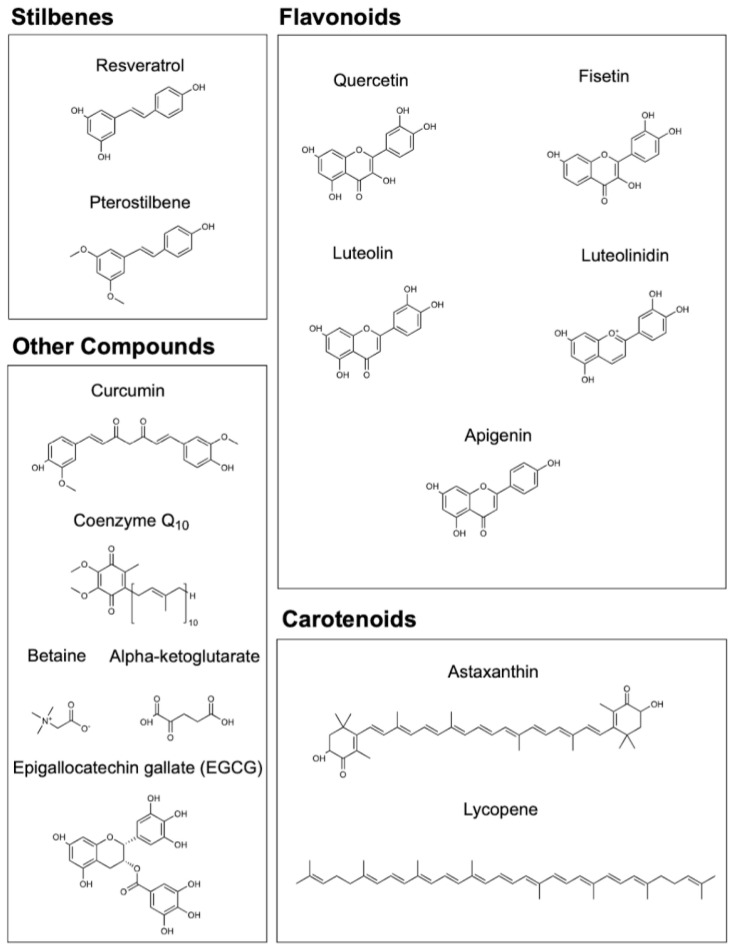
Chemical structures of the aforementioned nutraceutical compounds are organized into their respective categories.

Some of the most extensively studied nutraceuticals are the non-flavonoid phenolic stilbene compounds resveratrol and pterostilbene [87] (Figure 2). These compounds are naturally occurring in grapes and berries and possess a plethora of anti-inflammatory and antioxidant characteristics, and even combat many age-related disorders such as diabetes and cancer [88]. Clinical analyses have identified these stilbenes as safe and bioavailable substances with doses of resveratrol up to 5 grams and pterostilbene to 250 mg [89,90].

However, resveratrol and pterostilbene have been shown to increase lifespan only in specific preclinical models, were context-dependent, showed mixed results, and are debated [91]. Pterostilbene has been shown to improve antioxidant function and increase longevity by activating longevity-associated genes, namely SIRT2, and has varying effects in male and female *Drosophila* models with the same longevity-inducing results but differing mechanisms [92]. Pterostilbene exhibits an 80% increased bioavailability, compared to that of resveratrol at 20%, and possesses many of the same beneficial biologically modulating effects of resveratrol. In the context of many disease states and aging, pterostilbene functions by upregulating endogenous antioxidant enzymes such as superoxide dismutase (SOD) and glutathione reductase (GR) [93]. The bioavailability of pterostilbene is an important factor in modulating SIRT1, as resveratrol has a much lower bioavailability and is metabolized quickly in comparison. Co-administration of resveratrol and pterostilbene may serve to maximize total bioavailability and maximize the benefits of each [94]. In response to particulate matter-induced oxidative damage, pterostilbene is able to mitigate the inflammatory response by decreasing mediators such as cyclooxygenase 2 (COX2), mitogen-activated protein kinase (MAPK), and the collagen-degrading matrix metalloproteinase (MMP)-9 expression, preventing the keratinocyte aging processes [95,96,97,98]. Resveratrol provides skin health benefits through its anti-angiogenic and wound-healing properties [99,100].

While resveratrol may also have numerous other targets, experimental models have shown a close association with the SIRT1 pathway when investigating resveratrol dosage and supplementation [101,102]. Resveratrol has exhibited many health benefits but yielded mixed results when used for the treatment of disease specifically, and the role of resveratrol in the direct activation of SIRT1 has been heavily questioned. Resveratrol seems to be at least indirectly involved in the activation of SIRT1 to induce the beneficial effects of caloric restriction, which has been shown numerous times to induce lifespan extension [103]. The activation of SIRT1 along with AMPK and PGC1α in response to resveratrol is implicated, but despite this lack of clarity in interventional disease trials, resveratrol might still be considered an age-related metabolic modulator [104].

Resveratrol may also enhance neuroprotection by reducing neuroinflammation, thereby improving cognition and neuro-metabolic protection and decreasing the risk of age-related neurodegeneration. Resveratrol also possesses the ability to improve oxidative stress in the vasculature by reducing superoxide generation and increasing the antioxidant response of endothelial cells, thereby dissipating the age-related risk of cardiovascular disease, also initiated through the SIRT1-involved pathway. Anti-metastatic and anti-cancer properties are notoriously associated with resveratrol, as it helps suppress tumor proliferation and uncontrolled cellular division [105]. Interestingly, resveratrol has the ability to mimic caloric restriction (CR) in a similar fashion to that associated with the SIRT1 pathway to extend longevity. This occurs through several mechanisms. First, increasing the activity of telomerase and FoxO3a, a transcription factor associated with increased longevity. It also occurs through active regulation of Sirt1 (AROS). Finally, increasing Hu antigen R (HuR) and decreasing p53 can prevent cellular senescence but inhibit tumor suppression [106,107,108]. It has also demonstrated the ability to improve biological parameters of kidney aging [109] and improves arterial aging by reducing media thickness, inflammation, fibrosis, and oxidative stress through increasing SIRT1, AMPK, and PGC1α, SOD1, and SOD2 expression, as well as decreasing NADH oxidase 2 and 4 [110,111].

At low concentrations, resveratrol can inhibit cellular senescence; however, at higher concentrations, it exhibits complete senolytic activity [112]. Resveratrol can even exert an influence on reproductive aging, as seen in the postovulatory oocytes of mice. Rates of fertilization and blastocyst size increased in response to resveratrol administration, as well as upregulation of FoxO3a [113,114]. The cognitive effects of stilbene administration have gained more of a solid foundation in recent years, supporting its benefits in supplementation [115,116]. Cognitive impairment due to aging was attenuated by improved cognitive performance in older mice and decreasing levels of inflammatory mediators such as IL-1β and TNF-α in response to resveratrol [117], and aged mice that received resveratrol showed improvements in cerebrovascularity [118]. In response to a high-fat diet in rats, resveratrol or alpha-ketoglutarate (aKG) improved brain and metabolic aging profiles, notably through inflammatory response genes [119]. Witte et al., 2014, analyzed resveratrol administration at 200 mg daily in older adults to affect memory performance, hippocampal connectivity, and glucose metabolism. The results were that memory retention increased in the interventional group, along with increased functional connectivity in the frontal, parietal, and occipital regions of the hippocampus. Hemoglobin A1c (HB1Ac) and body fat decreased, implying lowered blood sugar, and the satiety hormone, leptin, increased. Both changes correlated with increased memory retention and increased functional connectivity. These changes were consistent with the benefits reported in previous studies, further demonstrating enhanced cognitive function and improved metabolic profiles through stilbene supplementation [120,121]. In the context of neurodegeneration and Alzheimer’s disease patients with mild impairment, resveratrol treatment resulted in a decrease in neuroinflammatory profile and MMP9 expression, but further studies are still required to validate the mechanism and efficacy in neurodegenerative disorders [122].

In the treatment of diabetes, resveratrol has been shown to improve oxidative and inflammatory microRNA profiles and reduce insulin resistance in diabetic humans [123]. Interestingly, resveratrol administration has been quite beneficial in ameliorating post-menopausal symptoms in older women. The risk of osteoporosis and improvements in non-osteoporotic post-menopause-related bone density were reported in response to 75 mg of daily resveratrol administration [124]. A 24-month crossover study in 125 postmenopausal women showed marked improvements in cognition, cerebrovascular function, and insulin sensitivity [125], and sustained cognitive, cardiovascular, and cerebrovascular benefits were reported over a 12-month study period in response to the same daily dosage, 75 mg, of resveratrol [126]. These results strongly implicate resveratrol ameliorating age-related dysfunction in post-menopausal women as a preventative measure.

Resveratrol combined with the senolytic flavonoid quercetin has also been shown to improve aging panels of the kidney in high glucose conditions by reducing the glycation precursor, methylglyoxal, and increasing senescence marker protein 30 (SMP30) expression [127]. Rat heart tissue has also shown improved inflammatory, apoptotic, and oxidative panels in response to resveratrol administration, particularly through the reduction of the inflammatory markers TNF-α and IL-gamma [128]. In particular, Ghanim et al. demonstrated that in response to a high-fat and high-carbohydrate meal, 100 mg of resveratrol and 75 mg of grape skin polyphenols were able to upregulate the binding activity of oxidative stress-response transcription factor nuclear factor erythroid 2-related factor (Nrf2) to detoxification target genes NAD(P)H Quinone Dehydrogenase 1 (NQO-1) and glutathione-S-transferase pi 1 (GST-pi1), as well as ameliorate the effects of inflammatory mediators such as the cluster of differentiation 14 (CD14), IL-1β, and Toll-like receptor 4 (TLR4), potentiating their anti-diabetic, anti-inflammatory, and anti-atherosclerosis effects [102,129]. Induced cartilage stiffening in response to ribose administration can also further be ameliorated by resveratrol as a result of decreased glycation and advanced glycation end products (AGE) because of the competitive collagen II binding activity of resveratrol and curcumin [130]. Resveratrol can further improve insulin sensitivity and skeletal aging through the SIRT1 pathway by reducing FoxO1-induced transactivation of pyruvate dehydrogenase kinase 4 (PDK4), increasing muscular metabolism integrity [131,132].

Furthermore, resveratrol and pterostilbene’s effects might be heightened when used in combination with NMN supplementation to produce optimal results as a cleverly tailored nutraceutical orthomolecular approach to delaying or even reversing the signs of aging [133]. Age- and risk-related biological markers for certain diseases can also be monitored in response to resveratrol and pterostilbene administration [134,135]. Coadministration of NMN and resveratrol resulted in increased NAD+ levels in the heart and skeletal muscle compared to NMN alone. NMN and ginsenoside coadministration resulted in higher NAD+ levels compared to NMN alone in the brain, heart, kidney, and lung tissues, further supporting the dual supplementation approach to maximize NAD+ levels in the body [136]. Resveratrol also retains the ability to activate NMNAT1, the NAD+ synthetic enzyme, to increase NAD+ levels by up to five times, providing a large substrate store for SIRT1 activation to promote longevity-enhancing pathways [137]. NR and pterostilbene co-administration additionally exhibit a two-dose, dose-dependent increase in NAD+ levels in acute kidney injury patients [138], and repeated doses of NR and pterostilbene also exhibit an increase in NAD+ levels of 40% and 90% (double dose) in humans with a consistent increase over the 8-week trial duration [139]. Resveratrol and quercetin are also able to promote cell protection and NAD+ levels along with NAD+ precursors by potentiating the cell rescue effects of said precursors in response to DNA damage [140]. Thus, by supporting the transient supplementation of NMN, stilbene combinations may maximize the potential for this longevity-based orthomolecular approach.

### 3.2. CoQ10

Coenzyme Q10 (CoQ10; Figure 2), or ubiquinol (the oxidized form of ubiquinone), participates in the electron transport chain in the mitochondria, and low levels of CoQ10 are associated with diseases such as neurodegenerative disorders, diabetes, cancer, fibrosis, and cardiovascular diseases [141]. Supplementation with CoQ10 in the context of disease states aims to re-establish antioxidant activity to ameliorate homeostatic disruptions [142]. CoQ10 has protective cardiovascular qualities and can result in improvements in hyperglycemia, hypertension, oxidative stress, and risk of cardiac events [143]. Endogenous biosynthesis of CoQ10 sharply decreases with aging, and enhanced longevity can be seen with higher mitochondrial levels of CoQ10. Skeletal muscle integrity in elderly individuals is associated with higher plasma CoQ10 content, and inflammatory factors such as TNF-α, IL-6, and C-reactive protein (CRP) levels can be reduced as a result of supplementation [144]. CoQ10 is also required for lipid integrity and protection of LDL oxidation against atherosclerosis [145]. Replenishing the age-related decline in levels of CoQ10 is imperative to reduce the risk of age-related diseases and lower the burden of age-inducing oxidative stress through nutraceutical supplementation [146]. CoQ10 supplementation, along with dietary interventions in elderly men and women, resulted in improved metabolic profiles, reducing metabolic and cardiovascular risk [147].

CoQ10 and NAD+ supplementation also portray a degree of synergy when analyzing maximum heart rate after exercise in chronic fatigue syndrome (CFS). Maximum heart rate was decreased, and reports of fatigue improved [148]. CFS shares some characteristics with aging and age-deregulated pathways, such as inflammation and oxidative stress, and CoQ10 and NADH supplementation were further shown to improve fatigue and levels of NAD+/NADH, CoQ10, ATP, citrate synthase, and lipoperoxides [149].

The antioxidant, anti-inflammatory, and age-related effects of CoQ10 make it a highly appealing supplement to include in the comprehensive orthomolecular approach to battling the biological process of aging, particularly in the context of supporting NAD+ levels. Further studies are required to show, for instance, the beneficial synergistic effects of NAD+ precursor supplementations in combination with CoQ10.

### 3.3. Betaine

Trimethylglycine, known as betaine, was originally derived from the beetroot plant (Figure 2). It has osmoprotectant and anti-inflammatory properties. Betaine, along with methionine and choline, is one of the primary methyl group donors involved in DNA methylation, and DNA methylation rates have been directly correlated with available access to methyl donors [150]. Betaine also suppresses numerous inflammatory expression profiles, such as TNF-α, COX2, and NF-kB activity [151]. The effects of betaine are implicated in postponing aging-related pathologies by promoting optimal lipid and glucose metabolism, inhibiting inflammatory transcription processes, and reducing cellular ER stress [152].

Interestingly, betaine and choline levels are influenced by the degradation of the NAD+ precursors, namely NAM, which consumes more betaine than choline. This potentially compromises the availability of a healthy pool of methyl donors [153]. The high burden of NAM degradation on betaine levels and epigenetic changes warrants adequate supplementation of methyl donors during concomitant NAD+ precursor administration, particularly NAM [154], but the effects of NMN or NAD+ conversion on methylation levels have not been explored. Concurrent supplementation of NMN, NAD+, or other precursors alongside betaine may prevent the proposed decline in betaine levels, maintaining proper methylation health and function.

### 3.4. Flavonoids: Quercetin, Fisetin, Luteolin/Luteolinidin, and Apigenin

Numerous flavonoids have also been shown to have a host of health benefits along with strong senolytic activity, such as fisetin, quercetin, luteolin/luteolinidin, and apigenin.

Fisetin and quercetin are potent anti-cancer agents and have been shown to have anti-tumor activity through calcium-induced tumor apoptosis along with improving cancer-related inflammatory profiles [155].

Fisetin has been shown to be a potent senolytic in older and progeroid models of mice, as well as in murine and human adipose tissue (Figure 2). Administration of fisetin improved lifespan and tissue homeostasis in mice [156]. Senolytic effect is a crucial characteristic of an interventional dietary supplement, and fisetin has been shown to perform as one of the strongest among numerous other flavonoids and compounds. The safety and efficacy of fisetin are currently being investigated in reducing inflammation and improving walking speed in frail elderly individuals in Phase 2 randomized clinical trials (NCT03675724, NCT03430037). Interestingly, fisetin also portrays a degree of interrelatedness to NAD+/NADH age-related pathology through its activation of SIRT1 by inducing mitochondrial fragmentation in a similar fashion to treatment with NAM and an increased NAD+/NADH ratio [157]. These findings point to the possibility that fisetin may have geroprotective effects in the context of the NAD+/SIRT1/CD38 pathway, but further experiments must be conducted to see measurable and explicit effects on the biochemical longevity axis.

Quercetin, another flavonoid with striking structural similarity to fisetin (Figure 2), is also considered a senolytic with many benefits in the realm of cardiovascular disease, neurodegeneration, inflammation, oxidative stress, cancer, and diabetes. Quercetin has been shown to be a geroprotective agent for in vitro models of premature aging [158,159]. Quercetin also exhibits attractive properties as a supplemental ingredient in the NAD+ boosting approach. Along with the previously mentioned apigenin, quercetin has been shown in the same experiment to be a potent CD38 inhibitor to maintain the integrity of NAD+ stores and act as a protective agent against metabolic disorders [160]. Quercetin can potently initiate SIRT1′s intermediary action to result in anti-inflammatory and antioxidant effects in diet-induced atherosclerosis rat models [161]. NAD+/NADH balance is also further affected by quercetin’s ability to oxidize NADH and decrease the NADH/NAD+ ratio, where larger concentrations of the flavonoid stimulated the Krebs cycle, likely due to an increase in available NAD+ [162]. Nevertheless, quercetin earns a role in the modulation of the NAD+/SIRT1/CD38 axis due to its effects on altering the NAD+/NADH ratio, SIRT1 activation, and CD38 inhibition.

Luteolin, another flavonoid with similar health benefits as other geroprotectors, also has strong anti-inflammatory capabilities in relation to skin aging, skin disease, skin cancer, and cognition [163,164] (Figure 2). Luteolin is implicated in CD38 mechanisms along with apigenin and quercetin (and other compounds, such as luteolinidin and kuromanin) and has been shown to be a potent inhibitor of CD38, ultimately leading to an increase in available NAD+ [165]. Luteolinidin also exhibits myocardial protective attributes by stabilizing NADPH and mitigating the ischemic injury-induced upregulation of CD38 [166]. Flavonoids may have further longevity-promoting effects through the clearance of cellular senescence during concurrent administration of NAD+ supporting compounds, and further compounds may be tested on their activity on NAD+/NADH ratios and sirtuin activation [167].

The flavonoid apigenin is derived from plants, such as parsley and chamomile, and possesses highly anti-inflammatory, antioxidant, and anti-carcinogenic properties (Figure 2). It can reduce COX2, IL6, and TNF-α in response to bacterial toxins and lipopolysaccharide (LPS) [168]. Upregulation of antioxidant enzymes such as SOD, glutathione peroxidase (GPX), and glutathione reductase (GR) is also evident in response to lower dose apigenin administration [169]. Apigenin achieves its anti-carcinogenic activity by downregulating key factors in cancer pathways and sensitizing tumor cells to chemotherapeutic interventions [170]. In the context of esophageal cancer, IL-6 transcription is inhibited in response to apigenin as an intervention [171], and apigenin also exhibits anti-leukemic activity in vivo and in human cells by inducing apoptosis [172]. Apigenin can further attenuate metabolic complications leading to the risk and characteristics of metabolic disorders, such as metabolic syndrome, atherosclerosis, and diabetes [173]. Apigenin reduces the expression of COX2 and can also produce anti-inflammatory effects in the context of cardiovascular disease, neurodegeneration, diabetes, and cancer [174]. Apigenin supplementation has anti-obesity effects, with marked reductions in visceral adipose tissue in high-fat-fed mice, and can preserve skeletal muscle in obese mouse models of obesity-induced skeletal muscle atrophy [175,176]. Apigenin also possesses the ability to ameliorate deleterious age-related changes in vascular endothelial function and structure due to oxidative stress [177]. The anti-inflammatory and anti-cancer properties of apigenin make it appealing to utilize in the context of healthy aging and geroprotection [178].

The most notable property of apigenin in the context of NAD+ supplementation is the involvement with the SIRT1 NAD+ and CD38 axis. Apigenin administration increases the activation ratio of SIRT1, NAD+, NAD+/NADH and strongly inhibits CD38, thereby enhancing endogenous NAD+ levels. The resulting increase in SIRT1 reduced cellular senescence as a result of oxidative stress [179]. Apigenin is a potent inhibitor of CD38, and knockout models have shown resistance to metabolic disorders and exhibit increased NAD+ levels. Inhibition of CD38 in obesity models led to an enhanced metabolic profile, increased NAD+ levels, and decreased protein acetylation, possibly due to enhanced SIRT1 activity due to higher NAD+ availability [160]. CD38 inhibition through apigenin administration additionally restores mitochondrial function in response to oxidative stress and increases the NAD+/NADH ratio and SIRT3 activation [180]. Apigenin serves as a crucial component in the comprehensive approach to restoring age-related depletion of NAD+ levels through its strong inhibition of CD38, the degrading enzyme of NAD+. Through its CD38 inhibitory activity, apigenin becomes a valuable interventional agent when maximizing the effects of increasing NAD+ availability in the body through NMN supplementation.

### 3.5. Carotenoids: Astaxanthin and Lycopene

Astaxanthin is a potent antioxidant carotenoid compound that offers an array of health benefits by mitigating reactive oxygen species (ROS) and supporting mitochondrial integrity [181] (Figure 2). Unsurprisingly, astaxanthin is highly efficacious in utilizing SIRT1 to achieve longevity-promoting effects in many experimental models. Astaxanthin was shown in vivo to soothe oxidative stress in response to brain injury by upregulating the expression of Nrf2 and SIRT1 while concurrently reducing the expression of pro-apoptotic factors, potentially reducing the risk of neuronal death events post-injury [182]. The effects of a high-fat-induced diet on cardiac and fibrotic damage can also be ameliorated by astaxanthin through the upregulation of SIRT1, inhibition of inflammatory cellular mobility, and reduced incidence of collagen deposition leading to fibrosis post-injury [183,184]. Astaxanthin can additionally protect renal tissue post-injury through its upregulation of SIRT1 [185]. The most supportive finding of astaxanthin’s ability to boost NAD+ availability was found by Zhang et al., where NMN, astaxanthin, and blood orange extract were utilized to successfully increase NAD+ in aging zebrafish, exhibiting superiority in raising NAD+ levels compared to supplementation of just NR, NR and astaxanthin, and even NR and pterostilbene [186]. This study can serve as a basis for future studies of supplements to hone in on effective and precise dosages and synergistic combinations to maximize the effect of NAD+ boosting approaches in humans.

Lycopene, another red-pigmented carotenoid, also possesses strong antioxidant and anti-inflammatory characteristics (Figure 2). Oxidative stress and the intrinsic aging process can lead to plasma lycopene depletion and impaired absorption, and supplementation has been shown to improve physical performance, osteoporosis, and skin aging [187]. Interestingly, lycopene activates SIRT1 to impart muscle angiogenesis and reverse insulin resistance in models of age-related vascular decline [188]. In vivo and in vitro models of D-galactose-induced aging were ameliorated in a superiority trial with astounding results in response to NMN and lycopene combination therapy, with increases in SOD; GSH-px, GPX, CAT, T-AOC, and evidence of senolytic abilities. Nrf2 was upregulated, and in vivo models showed enhanced cognition compared to NMN alone [189].

### 3.6. Curcumin

Curcumin has earned a role as a potent senolytic, along with the previously mentioned flavonoids (Figure 2). It has been implicated in improving senescence in age-related pathologies and has even exhibited modulatory effects on longevity-related pathways, such as the mTOR and FoxO [190]. In the context of neurodegeneration, curcumin has been shown to upregulate SIRT1 and, in the cardiovascular context, AMPK [191]. Anti-cancer, migration, and anti-angiogenesis activity can be observed in response to curcumin administration in head and neck squamous cell carcinoma experimental cell models [192]. Six-week curcumin supplementation has been shown to improve antioxidant and aerobic capacity in human runners, along with an increase in SIRT3 [193]. The connection between curcumin and sirtuins is evident, but the utilization of curcumin along with NAD+ boosting molecules must be first demonstrated in future superiority trials involving combination therapies.

### 3.7. Alpha-Ketoglutarate

Alpha-ketoglutarate (aKG; Figure 2) is a crucial metabolic intermediate that is involved in the Krebs cycle and is implicated in the process of aging [194]. Along with other geroprotectors that inhibit the TOR pathway, AKG is able to additionally inhibit ATP synthase to extend the lifespan in *C. elegans* in a fashion similar to caloric restriction [195]. Offering metabolic and antioxidant benefits [196], aKG produces lifespan-extending benefits which are additionally apparent in mice, and recent pilot clinical trials in humans have shown that Rejuvant, a novel formulation of aKG, was successful in reducing the biological age of study participants [197,198]. However, data involving aKG and NAD+ interplay are lacking and must be explored further.

### 3.8. Epigallocatechin Gallate

The polyphenol epigallocatechin gallate (EGCG; Figure 2), found in green tea, has been touted to retain neuroprotective, antioxidant, and anti-inflammatory effects and is currently being analyzed for its role in ameliorating numerous different diseases [199]. It has been shown to increase rat lifespan in response to oxidative stress [200]. However, the role of EGCG in SIRT1 modulation is unclear, as certain studies see upregulation of SIRT1 in response to administration [201], but others observe downregulation, particularly in cancerous cells [202,203]. EGCG may behave differently on SIRT1 depending on the context and which longevity factors must be upregulated to mitigate oxidative stress, and the effects of EGCG on NAD+/NADH ratio must be further observed.

## 4. Conclusive Remarks

**Figure 3:**
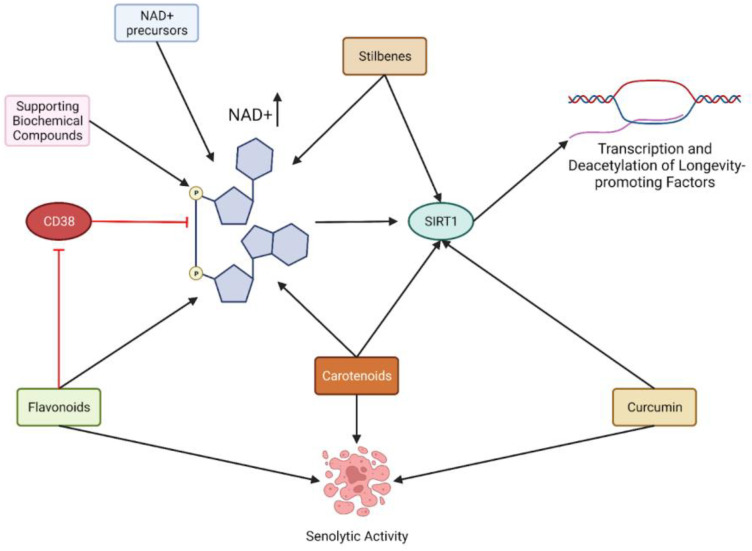
Hypothesized model of supplementing NAD+ precursors with other NAD+ enhancing geroprotectors. In addition to NAD+ precursors to raise NAD+ levels and enhance SIRT1 activity, stilbenes are able to support NAD+ levels and further activate SIRT1. Many flavonoids retain senolytic and CD38-inhibitory activity and can further innervate NAD+ stores. Curcumin and carotenoids retain similar properties in addition to SIRT1 activation. This interacting web of support may result in higher NAD+ stores than precursor-alone administration, producing longevity-promoting transcriptional benefits.

Having established the benefits of numerous ingredients involved in longevity-related pathways, a clearer picture emerges of the entangled molecular web of the NAD+/SIRT1/CD38 axis in the context of healthy aging supplementation (Figure 3). Many ingredients on their own show numerous benefits in the realm of many age-related disorders, such as neurodegeneration, cardiovascular and metabolic disease, diabetes, cancer, frailty, sarcopenia, hearing loss, osteoarthritis, osteoporosis, and ocular disorders [204]. CD38 inhibitors may become a focus for developing or screening new compounds, which may imply longevity induction in vivo and, ultimately, in humans. CD38-inhibitor 78c is one compound that has yet to enter human interventional trials but has shown marked improvements in longevity, physical performance, and metabolic profile in mice [205].

A beneficial direction may be to further look into other senolytics that ameliorate numerous hallmarks of aging. Further clarification of SIRT1 activity in relation to downstream effects implicated in longevity extensions shown in experimental models is required, particularly in humans, to determine the maximal effect of innervation through nutraceutical interventions. Testing superiority trials of NMN or NR in combination with other ingredients compared to NMN or NR alone, geroprotectors, senolytics alone, or other NAD+ precursor molecules will prove vital in maximizing the effects of each ingredient along with maximal NAD+ boosting potential. The potential of generated knowledge from further analyzing these relationships within the context of human aging may also offer insight into other pathways that may be implicated in longevity but may yield mixed results in the context of the fine balance for tumor suppression, senescence, and senolytic activity. Subsequently, avenues to target the pathology of aging and age-related diseases to increase healthspan may arise upon further exploration of other geroprotectors, particularly within clinical trials.

Due to the lack of superiority trials analyzing combination therapies and potential molecular synergies, a call for further RCTs is highly warranted in the field of healthy aging orthomolecular medicine and geroprotective nutrition. By screening for compounds that exhibit an effect on aging-related molecular pathways, innovative formulations of geroprotective ingredients can be achieved. Perhaps the maximum benefit can be derived not simply from one compound but from an ingredient profile that incorporates multiple compounds that work together to achieve a synergistic effect. If a formulation can function to boost NAD+ levels and SIRT1 activity and/or reduce CD38 activity, this will optimize lifespan and healthspan via extension supplements. Further, the more pathways that are affected, the more broadly these supplements can be applied to have a positive impact on the health and aging of multiple bodily systems. This will expand the application of these supplements, thereby broadening the populations that they may benefit.

## Figures and Tables

**Table 1:** Various clinical trials (completed and ongoing) detailing the use of NMN and other NMN derivates to improve health, metabolic markers, and disease parameters.

Clinical Trials	Compound of Interest
Nicotinamide Mononucleotide Increases Muscle Insulin Sensitivity in Prediabetic Women [82]	NMN
Effect of Oral Administration of Nicotinamide Mononucleotide on Clinical Parameters and Nicotinamide Metabolite Levels in Healthy Japanese Men [83]	NMN
Nicotinamide Mononucleotide Supplementation Enhances Aerobic Capacity in Amateur Runners: a randomized, double-blind study [77]	NMN
Effect of 12-Week Intake of Nicotinamide Mononucleotide on Sleep Quality, Fatigue, and Physical Performance in Older Japanese Adults: a randomized, double-blind placebo-controlled study [79]	NMN
Safety Evaluation of Beta-nicotinamide Mononucleotide Oral Administration in Healthy Adult Men and Women [84]	NMN
The Efficacy and Safety of Beta-nicotinamide Mononucleotide (NMN) Supplementation in Healthy Middle-aged Adults: a randomized, multicenter, double-blind, placebo-controlled, parallel-group dose-dependent clinical trial [85]	NMN
A Multicenter, Randomized, Double-Blind, Parallel Design, Placebo-Controlled Study to Evaluate the Efficacy and Safety of Uthever (NMN Supplement), an Orally Administered Supplementation, in Middle-Aged and Older Adults [73]	NMN
MIB-626, an Oral Formulation of a Microcrystalline Unique Polymorph of Beta-Nicotinamide Mononucleotide, Increases Circulating Nicotinamide Adenine Dinucleotide and its Metabolome in Middle-Aged and Older Adults [86]	MIB-626
Phase 2a MIB-626 vs. Placebo COVID-19 (NCT05038488)	MIB-626
Effect of Oral NAD+ Precursors Administration on Blood NAD+ Concentration in Healthy Adults (NICO) (NCT05517122)	NAM, NR, and NMN
Effect of NMN Supplementation on Organ System Biology (VAN) (NCT04571008)	NMN
Pharmacodynamics and Tolerance of Nicotinamide Mononucleotide (NMN, 400mg/Day) in Healthy Adults (NCT04862338)	NMN
Study to Evaluate the Effect of Nicotinamide Mononucleotide (NMN) As an Adjuvant to Standard of Care (SOC) On Fatigue Associated with Covid-19 Infection (NCT05175768)	NMN
Nicotinamide Mononucleotide in Hypertensive Patients (NCT04903210)	NMN
Safety and Pharmacokinetics of Nicotinamide Mononucleotide (NMN) in Healthy Adults (NCT04910061)	NMN
Effect of NMN (Nicotinamide Mononucleotide) on Polycystic Ovary Syndrome (NMN) (NCT05305677)	NMN
Effect of NMN (Nicotinamide Mononucleotide) on Diminished Ovarian Reserve (Including Premature Ovarian Insufficiency) (NCT05485610)	NMN
Effect of NMN on Muscle Recovery and Physical Capacity in Healthy Volunteers with Moderate Physical Activity (NCT04664361)	NMN

## Data Availability

Not applicable.
